# Integrating diverse genomic data using gene sets

**DOI:** 10.1186/gb-2011-12-10-r105

**Published:** 2011-10-21

**Authors:** Svitlana Tyekucheva, Luigi Marchionni, Rachel Karchin, Giovanni Parmigiani

**Affiliations:** 1 Department of Biostatistics and Computational Biology, Dana-Farber Cancer Institute, 450 Brookline Avenue, Boston, MA 02115, USA; 2 Department of Biostatistics, Harvard School of Public Health, 677 Huntington Avenue, Boston, MA 02115, USA; 3 Department of Oncology, Sidney Kimmel Comprehensive Cancer Center, Johns Hopkins University, 1550 Orleans Street, Baltimore, MD 21231, USA; 4 Department of Biomedical Engineering, Institute for Computational Medicine, Johns Hopkins University, 3400 N. Charles Street, Baltimore, MD 21218, USA

## Abstract

We introduce and evaluate data analysis methods to interpret simultaneous measurement of multiple genomic features made on the same biological samples. Our tools use gene sets to provide an interpretable common scale for diverse genomic information. We show we can detect genetic effects, although they may act through different mechanisms in different samples, and show we can discover and validate important disease-related gene sets that would not be discovered by analyzing each data type individually.

## Background

The increasing affordability of high throughput genome-wide assays is enabling the simultaneous measurement of several genomic features in the same biological samples. Cancer genome projects have been at the forefront of this trend, and have faced the challenge of integrating these diverse data types [1,2], including RNA transcriptional levels, genotype variation, DNA copy number variation, and epigenetic marks. Annotated collections of gene sets, capturing established knowledge about biological processes and pathways, have proven an essential tool for integration. Examples of these sets include chromosomal locations, signaling and metabolic pathways, transcriptional programs, and targets of specific transcription factors. Because one can make inferences about the importance of a given gene set using several different genomic data types, gene set analysis provides a direct and biologically motivated approach to analyzing these data types in an integrated way. A widely used public collection of gene sets is the Molecular Signatures Database (MSigDb) [3]. A comprehensive list of conventional tools for gene set analysis for a single data type is given in Ackermann *et al*. [4]. Many of these approaches are implemented in the extensively used statistical computing environment R/Bioconductor [5].

The gene set perspective makes sense both biologically and statistically. First, small differences in the functions of multiple genes in the same set may not be detectable at the single gene level, but can add to create larger differences at the gene set level. This increases the power for detecting real biological differences. Second, a single hit on a given pathway may be sufficient to generate a phenotypic difference. If this hit can occur in any of several components in the pathway, individuals with the same phenotype may show variability in the specific genes that are hit, but show a more consistent pattern at the pathway or gene set level [1,6]. Importantly, even when a difference at the single gene level can be detected, its biological importance may depend on the states of other interacting genes and gene products.

Cancer genomes contain point mutations, insertions, deletions, translocations, methylation abnormalities, and copy number (CN) and expression changes not seen in normal tissues. In some cancers, such as glioblastoma multiforme (GBM), different genes involved in pathways involving TP53, phosphoinositide 3-kinase (PI3K), and RB1 are altered in different patients, and, importantly, these might be altered via different mechanisms [1], such as point mutations and CN changes. Therefore, taking into account multiple data types should improve our ability to detect gene sets associated with a phenotype.

In recent large-scale cancer genome studies [1,6,7] preliminary integration approaches have been successfully applied; however, these approaches have been tailored to specific contexts. A general, a scalable and rigorous statistical framework has not yet been developed. In this article, our goal is to fill this gap. To this end, we introduce, compare, and systematically evaluate two alternative set-based data integration approaches. The first approach is based on computing model-based gene-to-phenotype association scores for each gene using all data types together, followed by gene set analysis of these scores. We term this the integrative approach. The second is to perform separate conventional gene set analyses for each data type, and then derive a consensus significance score using a meta-analytical approach.

## Results

### Overview

We present both novel data analyses and controlled simulations. First, we jointly examine gene expression and CN variation data about glioblastoma multiforme tumors from The Cancer Genome Atlas (TCGA) [2], and detect differences in the Wnt, glycolysis and stress pathways that appear relevant to differences between short- and long-term survivors. We also validate these findings using independent samples from the NCI Repository for Molecular Brain Neoplasia Data (Rembrandt) [8]. To provide a rigorous counterpart to these results we perform extensive simulations. These show that the integrative approach does enable the discovery of disease-related gene sets that would not be discovered when each data type is analyzed individually using current approaches. Discoveries remain reliable also when several features are highly noisy.

### The Cancer Genome Atlas glioblastoma multiforme study

We consider TCGA glioblastoma data [2] of four types: two gene expression measurements (E1, E2) and two CN measurements (C1, C2), described in Materials and methods. To discover gene sets important in GBM survival we use an extreme discordant phenotype design [9] with a total of 95 subjects. GBM patients with a survival time shorter than the lower quartile (190 days) are labeled short-term survivors (STSs), and those with a survival time longer than the upper quartile (594 days) long-term survivors (LTSs). Such grouping enhances signal relevant to survival. We used gene sets from the MSigDb canonical pathways.

First, we consider genes that are measured in all data types (genes that are measured only in a subset of platforms are filtered out), and use a competitive gene set test (see Materials and methods), comparing genes within a set to the remainder of the annotated genes. The 30 top sets discovered by the integrative approach are reported in Table 1. If we consider the top 30 sets, we discover 12 gene sets that are not discovered by any of the standard single-data-type analyses. The majority of these sets are related to metabolic processes. Six are involved in sugar-related metabolic processes and energy production, and two (the curated streptomycin biosynthesis pathway, and its KEGG (Kyoto Encyclopedia of Genes and Genomes) counterpart, hsa00521) are identified as a result of genes shared with the sugar metabolism group (six out of eight genes in the streptomycin biosynthesis set are paralogs of genes in the glycolysis pathway).

**Table 1:** *P*-values for top 30 gene sets discovered by the integrative method using the competitive gene sets test

Pathway	E1	E2	C1	C2	INT
AMINOSUGARS_METABOLISM*	0.0132	0.4429	0.4686	0.0085	0.0008
**STREPTOMYCIN_BIOSYNTHESIS**	0.1180	0.0822	0.8774	0.1301	0.0024
STARCH_AND_SUCROSE_METABOLISM*	0.0163	0.0457	0.6400	0.7045	0.0048
TRANSLATION_FACTORS	0.0006	0.0007	0.0700	0.0505	0.0092
HSA00860_PORPHYRIN_AND_CHLOROPHYLL_METABOLISM	0.0034	0.1760	0.1306	0.1287	0.0098
GLYCOLYSISPATHWAY*	0.1321	0.2590	0.1119	0.0716	0.0105
HSA00624_1_AND_2_METHYLNAPHTHALENE_DEGRADATION	0.0157	0.0037	0.3096	0.4940	0.0115
HSA03050_PROTEASOME	< 10^-4^	0.0021	0.7431	0.9031	0.0123
**HSA00521_STREPTOMYCIN_BIOSYNTHESIS**	0.1243	0.1364	0.9467	0.2227	0.0124
HSA00530_AMINOSUGARS_METABOLISM*	0.0123	0.2232	0.8209	0.2205	0.0130
FRUCTOSE_AND_MANNOSE_METABOLISM*	0.2724	0.6586	0.2115	0.0618	0.0140
**STRESSPATHWAY**	0.3783	0.0589	0.0636	0.0533	0.0175
HSA00500_STARCH_AND_SUCROSE_METABOLISM*	0.0328	0.3084	0.7397	0.8215	0.0188
**HSA00740_RIBOFLAVIN_METABOLISM**	0.3108	0.1873	0.6021	0.1767	0.0198
PORPHYRIN_AND_CHLOROPHYLL_METABOLISM	0.0444	0.1849	0.3903	0.0703	0.0206
**HSA00603_GLYCOSPHINGOLIPID_BIOSYNTHESIS_GLOBOSERIES**	0.3816	0.4798	0.2583	0.1846	0.0230
KREBS_TCA_CYCLE	0.3707	0.0663	0.3670	0.0117	0.0246
IL10PATHWAY	0.0391	0.0166	0.1396	0.0650	0.0263
BLOOD_GROUP_GLYCOLIPID_BIOSYNTHESIS_LACTOSERIES	0.0070	0.3230	0.4054	0.6334	0.0268
HSA00642_ETHYLBENZENE_DEGRADATION	0.0263	0.0198	0.1641	0.4937	0.0306
FEEDERPATHWAY*	0.1347	0.2727	0.2331	0.4541	0.0312
GALACTOSE_METABOLISM*	0.1987	0.2374	0.5055	0.0538	0.0333
CYTOKINEPATHWAY	0.0238	0.0439	0.1226	0.2484	0.0400
**WNTPATHWAY**	0.8890	0.2008	0.3227	0.0549	0.0402
HSA00051_FRUCTOSE_AND_MANNOSE_METABOLISM*	0.5893	0.7157	0.3759	0.0078	0.0408
GLYCOSAMINOGLYCAN_DEGRADATION*	0.0135	0.4210	0.0348	0.0208	0.0464
GLUCONEOGENESIS*	0.1634	0.0849	0.7657	0.5843	0.0488
GLYCOLYSIS*	0.1634	0.0849	0.7657	0.5843	0.0488
PROTEASOMEPATHWAY	0.0015	0.0702	0.6308	0.7039	0.0497
HSA00052_GALACTOSE_METABOLISM*	0.2507	0.4202	0.5990	0.0354	0.0497

The sets discovered using integration but not using any of the single-data-type analyses are in bold font. An asterisk denotes sets related to sugar metabolic processes. Discoveries are sets whose *P*-value is < 0.05. E1 and E2, single data type analysis using expression data; C1 and C2, single data type analysis using copy number data; INT, integrative method.

This metabolic shift toward sugar metabolism is not surprising since it is known that cancer cells in general [10,11], and glioblastoma cells in particular [11], depend on the conversion of glucose to lactate in the presence of oxygen (Warburg effect [12]). It has also been shown that shutdown or down-regulation of the glycolysis pathway in glioblastoma is associated with cell death [13,14].

**Figure 1:**
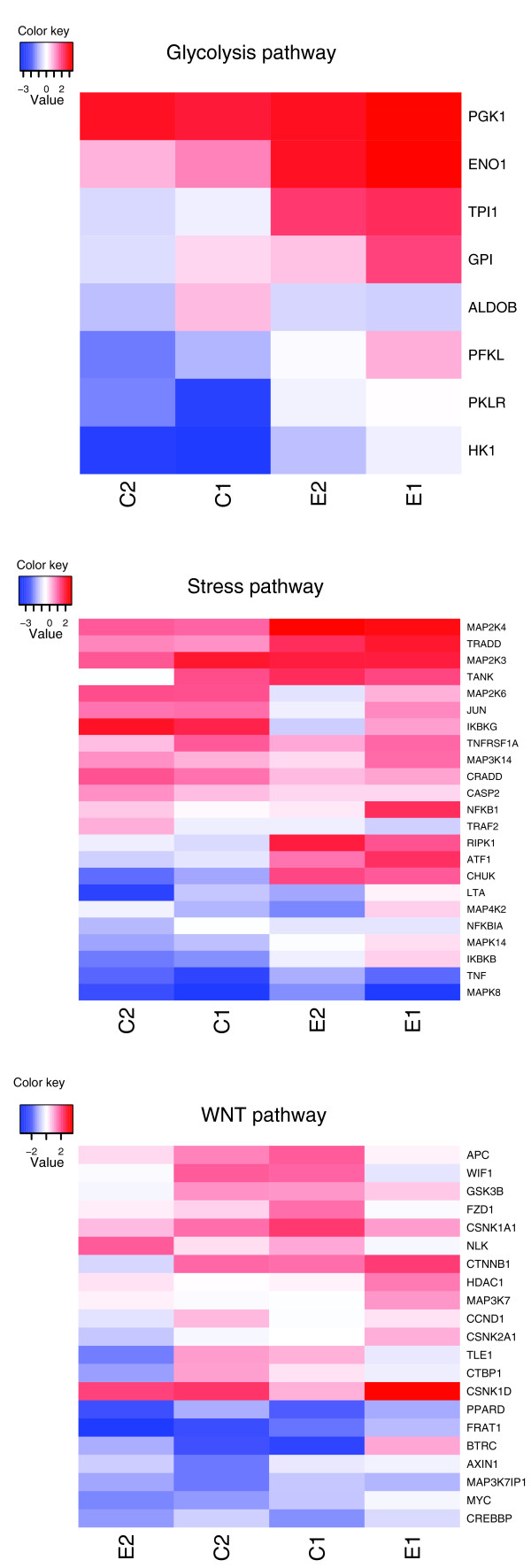
**Heatmaps of the two sample t-statistic from each data type between long- and short-term survival phenotypes**. Color keys larger than 1.9 or smaller than -1.9 approximate statistical significance of the difference. Positive t-statistic means higher average measurements for short-term survivors.

We find that mean measurements for glycolytic genes are, on average, larger in the STS compared to the LTS phenotype (Figure 1) and that there are more gene copies in STSs. Since reduced glycolysis (and sugar usage) promotes GBM cell death, we speculate that there might be an association between patient survival and efficient sugar metabolism, which is being detected by the integrative approach but missed by conventional analysis of each data type separately.

Necrosis and hypoxia are pathognomonic features of the highest-grade malignant gliomas and are thought to play a key role in the aggressive behavior of GBM, including invasiveness and chemo-resistance, through a variety of mechanisms [15-17]. The induction of the glycolytic pathway we have documented in STS patients is likely to represent an adaptive consequence of hypoxic conditions, mediated by genomic alteration and/or expression of hypoxia inducible factors (HIF1A and HIF2A), which have been shown to induce glycolytic genes [18], and recently to play a fundamental role in the expansion and maintenance of the GBM stem cell compartment [19,20].

The other metabolic processes related to gene sets identified in our analysis are riboflavin (vitamin B2) metabolism and the biosynthesis of glycosphingolipid. The involvement of the riboflavin pathways appears to be mostly driven by up-regulation of members of the myotubularin-related protein family (not shown), which act as phosphatases modifying cell membrane phospholipids. From this perspective, the concomitant enrichment of 'biosynthesis of glycosphingolipid', mostly determined by gene down-regulation at both the CN and expression level (not shown), may relate to an early observation that membrane lipid modifications occur during progression of human gliomas [21], and that glycosphingolipid profiles correlate with survival grading in human gliomas [22]. Intriguingly, a crucial role for the PI3K/AKT pathway in the regulation of lipid biosynthesis and signaling pathways was recently reported [23], linking our findings to the major molecular alterations in the PI3K pathway in GBM identified in TCGA [2].

Among the non-metabolic gene sets detected in our analysis, we highlight those associated with the stress and Wnt pathways. The stress pathway includes genes involved in TNF signaling through its receptors TNFR1 and TNFR2. Cellular responses to TNF encompass a wide range of processes, from induction of cell survival to apoptosis. The final outcome results from the modulation, integration and cross-talk of distinct signaling cascades that are initiated by TRADD (TNFR1-associated DEATH domain protein) and TRAF2 (TNF receptor-associated factor 2) [24,25]. The presence of this gene set in our analysis is mostly driven by increased expression/CN of pathway members in the STS phenotype (Figure 1). Factors involved in both survival and apoptosis are increased (that is, mitogen-activated protein kinase (MAPK) signaling pathway genes *NFKB1*, *TRADD*, *CRADD *(CASP2 and RIPK1 domain containing adaptor with death domain)). The only two genes with reduced expression in the STS group are those encoding TNF, which initiates the signaling, and MAPK8, which is required for TNF-alpha-induced apoptosis [26].

Although extensive evidence has been published describing the Wnt pathway's role in embryonic development, adult tissue homeostasis, and human disease, including cancer [27], little is know about the role of the Wnt pathway in GBM. However, recent findings have shown that promoter hypermethylation of Wnt pathway inhibitors occurs in GBM [28]. In our analysis, the relationship of the Wnt pathway to survival in GBM patients is driven by both increased and decreased expression/CN in the STS group (Figure 1) of genes encoding both inhibitors and activators of the Wnt pathway. 'Up-regulated' genes include central players of the pathway, specifically *CTNNB1 *(encoding β-catenin) and *GSK3B *(glycogen synthase kinase 3 beta). GSK3 phosphorylates the APC/AXIN1/CTNNB1 complex, and thus targets β-catenin for degradation. Wnt signaling activation determines GSK3 inhibition status, resulting in β-catenin stabilization, nuclear transfer and transcription activation. These results agree with the recent observation that GSK3 inhibition results in glioma cell death through a mechanism that depends on c-MYC activation, decreased NF-κB activity, and an alteration of intracellular glucose metabolism [29]. Even more interesting is that increases of GSK3 and NFKB1 in the STS group, at both the CN and expression level, are accompanied by decreased MYC levels (Figure 1).

To assess the sensitivity of our results to the choice of patients, we considered tertiles instead of quartiles, and we used a gene-level Cox regression model on the entire patient set. The results were very similar to those presented above (Tables S1 and S2 in Additional file 1).

Our gene-to-phenotype association scores are based on the difference of the deviances in the gene-level regression model. This metric depends on the number of variables included in the model. As the number of variables increases, the difference of the deviances will grow, even if the added variables are not truly correlated with the phenotype, and thus do not provide any additional biological signal. Therefore, competitive gene set tests cannot be used to analyze genes that are not measured for all data types because the genes that are measured on fewer platforms will get inferior rankings when compared to genes that might have the same strength of biological signals but are measured everywhere. However, restricting attention to the genes measured in all data types might lead to loss of some interesting biological information. We extended our analysis to the union of genes measured in at least one data type. To do this without biasing the results in favor of genes represented in multiple data types, we use a self-contained gene set test (see Materials and methods), comparing genes within each set to a null distribution based on those genes only. This test compares the observed data to an internal control based on the null distribution for the same set of genes: thus, the values for each gene under the null hypothesis account for the number of data types for which the gene is available, and the effect of the number of platforms on the association scores is properly controlled. The results are given in Table 2. The top sets share pathways with the competitive analysis, including sugar metabolic processes. Interestingly, the second most significant pathway (HSA04010_MAPK_SIGNALING) contains all the genes from the STRESSPATHWAY reported above. Smaller *P*-values and rearrangements in the list of top sets for the self-contained test, compared to the competitive one, are likely to result mostly from the different statistical meaning of the test (the two procedures test different null hypotheses).

**Table 2:** *P*-values for top 30 gene sets discovered by the integrative method using the self-contained gene sets test

Pathway	E1	E2	C1	C2	INT	INT for validation set
HSA04810_REGULATION_OF_ACTIN_CYTOSKELETON	0.0697	0.2250	< 10^-4^	< 10^-4^	< 10^-4^	0.0001
HSA04010_MAPK_SIGNALING_PATHWAY	0.1302	0.0746	< 10^-4^	< 10^-4^	< 10^-4^	< 10^-4^
HSA04060_CYTOKINE_CYTOKINE_RECEPTOR_INTERACTION	0.1422	0.0132	< 10^-4^	< 10^-4^	< 10^-4^	0.0147
HSA04310_WNT_SIGNALING_PATHWAY	0.2389	0.0039	< 10^-4^	< 10^-4^	< 10^-4^	0.0001
HSA04080_NEUROACTIVE_LIGAND_RECEPTOR_INTERACTION	0.1164	0.8695	0.0001	0.0005	< 10^-4^	0.0042
HSA00230_PURINE_METABOLISM	0.0034	0.0503	0.0000	0.0003	< 10^-4^	0.0022
TRANSLATION_FACTORS	0.0208	0.0155	0.0022	0.0052	0.0001	0.2711
HSA01030_GLYCAN_STRUCTURES_BIOSYNTHESIS_1	0.0781	0.0292	0.0004	0.0003	0.0001	0.0454
HSA04360_AXON_GUIDANCE	0.4293	0.1803	< 10^-4^	0.0106	0.0001	0.0001
GLUCONEOGENESIS*	0.0435	0.1010	0.0613	0.0106	0.0001	0.0409
GLYCOLYSIS*	0.0435	0.1010	0.0613	0.1065	0.0001	0.0409
HSA00500_STARCH_AND_SUCROSE_METABOLISM*	0.0448	0.2669	0.0140	0.0043	0.0001	0.0364
HSA04630_JAK_STAT_SIGNALING_PATHWAY	0.2975	0.0090	0.0022	0.0041	0.0002	0.0141
HSA04510_FOCAL_ADHESION	0.0423	0.0926	0.0042	0.0374	0.0002	10^-4^
MRNA_PROCESSING_REACTOME	0.1660	0.0062	0.0537	0.0004	0.0003	< 10^-4^
HSA05215_PROSTATE_CANCER	0.0534	0.3618	0.0013	0.0534	0.0004	0.0008
HSA01031_GLYCAN_STRUCTURES_BIOSYNTHESIS_2	0.0084	0.0076	0.0312	0.0141	0.0004	0.0926
HSA00240_PYRIMIDINE_METABOLISM	0.0013	0.0307	0.0022	0.0065	0.0004	0.0063
HSA04210_APOPTOSIS	0.1207	0.2064	0.0003	0.0001	0.0004	0.0021
HSA04620_TOLL_LIKE_RECEPTOR_SIGNALING_PATHWAY	0.1986	0.1228	0.0001	0.0109	0.0004	0.0006
GLYCOLYSIS_AND_GLUCONEOGENESIS*	0.0209	0.1407	0.0476	0.0112	0.0005	0.1808
HSA04660_T_CELL_RECEPTOR_SIGNALING_PATHWAY	0.0192	0.1176	0.0141	0.0027	0.0006	0.0661
HSA00051_FRUCTOSE_AND_MANNOSE_METABOLISM*	0.1565	0.2238	0.0314	0.0112	0.0006	0.2631
HSA00010_GLYCOLYSIS_AND_GLUCONEOGENESIS*	0.0515	0.1333	0.1276	0.0148	0.0007	0.0647
INTEGRIN_MEDIATED_CELL_ADHESION_KEGG	0.2779	0.1734	0.0017	0.0038	0.0007	0.0001
HSA04640_HEMATOPOIETIC_CELL_LINEAGE	0.0634	0.0676	0.0140	0.0009	0.0007	0.0026
GPCRDB_CLASS_A_RHODOPSIN_LIKE	0.3429	0.4710	0.0003	0.0483	0.0008	0.0449
HSA00350_TYROSINE_METABOLISM	0.0219	0.0421	0.0772	0.0085	0.0008	0.0648
HSA05221_ACUTE_MYELOID_LEUKEMIA	0.0697	0.3196	0.0213	0.0105	0.0009	0.0310
CALCINEURIN_NF_AT_SIGNALING	0.1302	0.1298	0.0205	0.0005	0.0010	0.1128

Asterisks indicate sets related to sugar metabolic processes. E1 and E2, single data type analysis using expression data; C1 and C2, single data type analysis using copy number data; INT, integrative method.

### Independent validation

We validated results by applying the same method to an independent set of glioblastoma samples from the Rembrandt database [8]. Because we could only acquire information on a relatively limited number of genes, we focused on validation of the top 30 sets emerging from the self-contained analysis. Despite smaller sample sizes, missing genes, and the availability of only two data types, the vast majority of the pathways discovered in TCGA show strong evidence of association with survival in our validation set (Table 2), and the directions of association are generally confirmed.

### Simulations

To generate data as realistic as possible, we began with the actual TCGA GBM data just described, reassigned phenotype labels at random, spiked in gene set differences, and asked each method to recover the set that had been spiked in. We used both synthetic non-overlapping gene sets and real chromosomal bands to capture both low and high within-set correlations. In each spike-in experiment we set a fraction γ of genes to be truly associated with the phenotype within a spiked-in set, and a strength β of the effect of the gene on the phenotype. We varied γ and β to generate alternative scenarios (see Materials and methods for details).

#### Synthetic gene sets

Our synthetic gene set collection mimics the size distribution of sets from the MSigDb canonical pathway collection [3]. Genes are assigned to sets at random, but so that sets in the collection do not share genes. Therefore, we do not expect two genes within a set to be more strongly correlated than any two genes not belonging to the same set.

**Figure 2:**
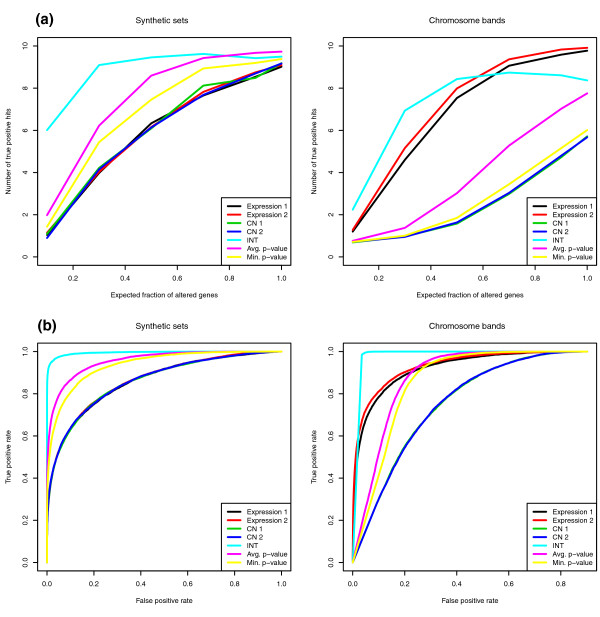
**Simulation results**. **(a) **Average number of discovered spiked-in sets among the top ten sets that are inferred to be enriched for genes discriminating between two phenotypes, against the expected fraction of the altered genes; β = 0.5. **(b) **Average ROC curves; β = 0.5, γ = 0.1. INT, integrative method.

We perform gene set analysis on all data types separately, using a standard methodology implemented in the *limma *package in Bioconductor [5,30]. We compare these to three multi-platform methods. For each method we rank sets by *P*-value, and select the top ten. We evaluate approaches by comparing the number of true positive hits among the top ten sets (Figure 2a). Results from the four individual data types are practically indistinguishable. All three multiple-data-type approaches outperform the single-data-type approaches. The integrative approach is significantly better for small values of γ, where subsets of altered genes are likely to be different across data types. In such settings the sensitivity of single-data-type methods will be relatively low, but the integrative approach enjoys increased sensitivity because the integrated gene-to-phenotype association score is sensitive to gene alterations in a single data type. This property is especially useful when genes in a certain set are altered by different biological mechanisms, and consequently measured via different data types. Both meta-analytical approaches perform similarly, and are outperformed by the integrative approach. This pattern of performance is similar for other values of β. All methods, as expected, perform better as β increases (not shown).

Figure 2b shows receiver operating characteristic (ROC) [31] curves from the same simulations to provide results that are independent of list size. The integrative approach tends to have the highest sensitivity and specificity across all *P*-value cutoffs. Accuracy results are summarized using areas under the ROC curves in Table 3. Importantly, the integrative approach generally shows less variability when compared to both single-data-type approaches and meta-analysis.

**Table 3:** Areas under the ROC curves for various simulation scenarios

		Method				
		
β	γ	E1	C1	INT	AvgP	MinP
Synthetic gene sets						
0.166	0.1	0.59 (0.1)	0.58 (0.09)	0.84 (0.07)	0.65 (0.09)	0.64 (0.09)
	0.5	0.83 (0.1)	0.79 (0.1)	0.98 (0.03)	0.91 (0.06)	0.89 (0.06)
	1	0.94 (0.07)	0.92 (0.07)	0.97 (0.03)	0.98 (0.03)	0.97 (0.03)
0.5	0.1	0.69 (0.09)	0.7 (0.09)	0.96 (0.03)	0.80 (0.07)	0.77 (0.07)
	0.5	0.94 (0.05)	0.94 (0.05)	1 (0.01)	0.99 (0.02)	0.98 (0.02)
	1	0.99 (0.02)	0.99 (0.01)	1 (0.01)	1 (0.01)	1 (0.01)
0.84	0.1	0.73 (0.08)	0.74 (0.08)	0.98 (0.02)	0.85 (0.06)	0.81 (0.07)
	0.5	0.97 (0.03)	0.97 (0.03)	1 (0)	1 (0.01)	0.99 (0.01)
	1	1 (0)	1 (0)	1 (0)	1 (0)	1 (0)
Chromosome bands						
0.166	0.1	0.65 (0.09)	0.55 (0.1)	0.78 (0.07)	0.63 (0.09)	0.64 (0.09)
	0.5	0.9 (0.08)	0.67 (0.09)	0.98 (0.02)	0.83 (0.06)	0.83 (0.06)
	1	0.98 (0.05)	0.72 (0.11)	0.95 (0.03)	0.93 (0.04)	0.93 (0.04)
0.5	0.1	0.72 (0.08)	0.60 (0.09)	0.90 (0.04)	0.70 (0.08)	0.70 (0.08)
	0.5	0.99 (0.02)	0.88 (0.04)	1 (0)	0.95 (0.02)	0.93 (0.02)
	1	1 (0.01)	0.94 (0.06)	1 (0)	0.99 (0.01)	0.99 (0.01)
0.84	0.1	0.76 (0.08)	0.62 (0.09)	0.93 (0.03)	0.74 (0.07)	0.74 (0.07)
	0.5	1 (0.01)	0.93 (0.03)	1 (0)	0.98 (0.01)	0.96 (0.02)
	1	1 (0)	0.99 (0.01)	1 (0)	1 (0)	0.99 (0)
Canonical pathways						
0.166	0.1	0.56 (0.08)	0.55 (0.08)	0.71 (0.07)	0.58 (0.08)	0.57 (0.07)
	0.5	0.70 (0.09)	0.70 (0.08)	0.87 (0.04)	0.78 (0.06)	0.76 (0.06)
	1	0.82 (0.08)	0.82 (0.07)	0.87 (0.05)	0.88 (0.04)	0.86 (0.05)
0.5	0.1	0.60 (0.08)	0.61 (0.08)	0.80 (0.06)	0.66 (0.07)	0.64 (0.07)
	0.5	0.81 (0.06)	0.82 (0.06)	0.92 (0.02)	0.89 (0.04)	0.87 (0.04)
	1	0.91 (0.04)	0.92 (0.03)	0.93 (0.04)	0.94 (0.02)	0.92 (0.02)
0.84	0.1	0.62 (0.08)	0.63 (0.08)	0.82 (0.05)	0.70 (0.07)	0.68 (0.06)
	0.5	0.84 (0.05)	0.85 (0.04)	0.93 (0.02)	0.91 (0.03)	0.90 (0.03)
	1	0.93 (0.02)	0.94 (0.02)	0.94 (0.02)	0.95 (0.02)	0.93 (0.02)

Mean (and standard deviation) of the area under the ROC curve for selected values of the signal strength (β), and expected fraction of altered genes (γ). E1, single data type analysis using expression data; C1, single data type analysis using copy number data; INT, analysis of four data types using the integrative method; AvgP and MinP, analysis of four data types using meta-analytical approach.

#### Chromosome bands

Our next gene set collection is defined by the chromosomal location of genes, and constitutes a partition of the genes measured. Here we expect mildly increased correlations within the sets for gene expression, as well as strong spatial correlations within sets, and between physically neighboring sets for the CN.

The performance of single-data-type approaches differs between expression and CN (Figure 2a). The strong spatial correlations between neighboring bands are inherited from the data that we use to construct our simulations. In the GBM tumor samples, amplifications and deletions tend to be large, sometimes spanning several chromosome bands, and are present only in subsets of patients. Because of that, the variability of the Wilcoxon test ranks for CN data tends to be much lower for neighboring bands than for randomly selected ones. Therefore, false positive calls for CN data tend to cluster by their ranks and chromosomal position. This explains the inferior performance when compared to expression data, which are not affected as much by spatial correlations. In contrast, mild correlations within the sets for expression data tend to aid discovery of the spiked-in sets. Both expression data types perform better for the chromosome band collection than for the synthetic sets, where genes within sets were not correlated. Since both CN data types tend to produce a large amount of false positive calls in the top ten list, the performance of integration methods may also deteriorate. The most affected method is meta-analysis by minimum *P*-value approach because when there is a disagreement between data types, the strongest signal is not necessarily correct. Averaging *P*-values leads to a better, but still unsatisfactory, performance. The integrative approach is affected the least by the poor performance of the autocorrelated CN data. It still performs best for small and intermediate values of γ, which are the most common. In practice, we may not know which data type is best at capturing the signal, in which case using an integrative approach provides a robust safe analytical plan. The ROC curves (Figure 2b) show that as a higher sensitivity is sought, the integration method still retains a substantial edge over the single-data-type methods. The performance of the meta-analytical methods improves and eventually approaches that of individual expression data, though it remains inferior to integration. Average areas under ROC curves are in given in Table 3.

#### MSigDb canonical pathways

The MSigDb canonical pathway collection differs from the previous two in that it is not a partition of genes. Thus, we expect to observe correlations within the sets, for biological reasons such as co-regulation, and between the sets, because they share common genes. In terms of our simulation study, an important issue is how to define a true positive call. When we spike-in preselected sets, we may also alter other sets that contain genes that were chosen to be associated with the phenotype. Recovering such unintentional spike-in sets should not necessarily be considered as a false positive. To account for this, we consider a discovered set to be a true positive if it shares at least 50% of the genes with the union of genes from the original spike-in sets.

All methods perform worse than before. This is partially explained by the difficulty of defining a true positive. Multi-data-type methods provide gains in performance of various magnitudes for almost all scenarios. For all β, with γ ≥ 0.9, the average *P*-value method marginally outperforms the integrative approach. The variability of the accuracy of the methods also slightly increases compared to the previous scenarios (Table 3).

### Novel discoveries

**Figure 3:**
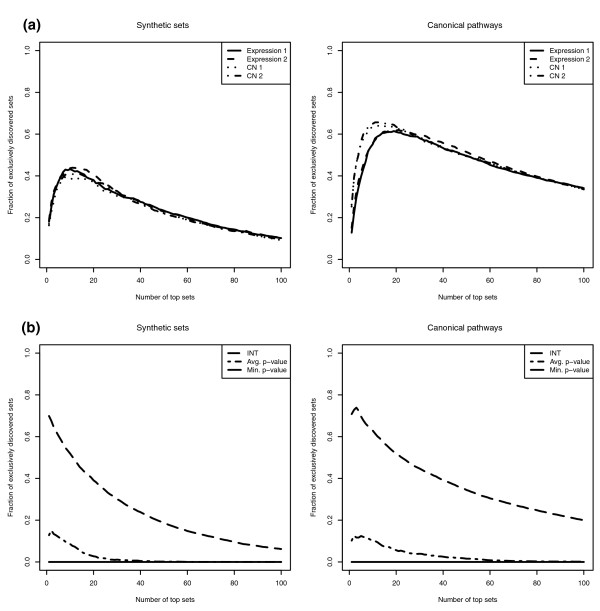
**True positive exclusively discovered sets**. **(a,b) **The average fraction of true positive exclusively discovered sets by each of traditional one-dimensional analysis (*EF *in Materials and methods section) (a) and integrative and meta-analytical methods (EF* in Materials and methods section) (b). β = 0.5, γ = 0.1. INT, integrative method.

Individual data types rank sets differently, discovering different sets for a given list size. Figure 3a shows the fraction of sets that are exclusively discovered by each data type. Figure 3b shows the additional sets discovered by the integrative and meta-analytical approaches but not by any of the single-data-type analyses (see Materials and methods for details). The meta-analytical approaches show minimal improvement over single-data-type analysis, while the integrative method discovers a large fraction of sets that are missed by all of the one-dimensional analyses. Statistically, this means that there is a large increase in the power to detect true effects for a given list size. In general, it cannot be guaranteed that a set identified by a single-data-type analysis will be necessarily identified using a model-based integrative approach. Such a property applies to the minimum *P*-value meta-analytical approach only when significance is held constant. However, our analysis of the ROC curves shows that our model-based integration approach has the most favorable combination of sensitivity and specificity.

## Discussion

We have developed and compared general approaches and specific tools to integrate data from genomic studies that measure multiple genomic features in the same subjects. Using simulations and data analysis, we demonstrate how integration can discover important patterns that would otherwise be missed.

Our methodology provides the greatest advantages when genes within a set are altered by different mechanisms. Consider a pathway of ten genes, where one is amplified, another is mutated, and a third is overexpressed. Single-data-type analysis and meta-analysis are likely to miss this pathway, while an integrative approach is poised to detect it. This does not come at the price of overemphasizing the pathway's importance when there is redundancy in the signal. For example, amplification and increased expression of a gene are likely to be correlated. The integrative approach recognizes that this is a single biological signal. Both expression and CN enter a single model for that gene, and the two measurements will only do marginally better as a pair than they would individually if they are highly correlated.

Our approach will adjust for varying reliability across data types: if the noise in a data type doubles, this data type would automatically contribute less to each gene-specific regression, and thus to the final result. Our approach could be easily modified to allow users to weight certain data types more heavily: for example, the gene-specific regression could be estimated using Bayesian methods, where the information on the biological importance of the data types is incorporated into prior distributions for the coefficients.

Application to other phenotypes and inclusion of covariates are also straightforward, by suitably choosing the type of regression used and the variables included. Cox regression could be used for survival data, linear regression for continuous phenotypes. Sliced inverse regression [32] could also prove useful, since it can be directly applied to both categorical and continuous phenotypes. Importantly, our approach entails no additional price in terms of multiple testing compared to one-dimensional analysis.

We operate within a two-stage framework. There are approaches for gene set analysis in other contexts that operate directly on the raw data - for example, on expression levels [33] or mutation counts [34]. These approaches have the advantage of allowing for a fuller treatment of uncertainty, some of which is lost by treating the regression-based scores as data. However, integration across platforms is greatly simplified by operating on gene summaries. Our initial assumption that each gene is measured once for each data type may be overcome by adding as many terms to the linear predictor as there are measurements for the gene in question, though additional investigation of this issue is needed. Other approaches achieve integration by building a network that exploit existing knowledge on the biological interactions within a pathway [35]. When available, this can be very valuable information. On the other hand, approaches considered here apply to far larger collections of gene sets - for example, positional gene sets or collections of previously described prognostic gene signatures - whose genes do not necessarily have direct functional relationships.

The integrative approach is not readily extensible to joint analysis of data when each data type is measured in a different set of patients. However, for these studies, meta-analytical approaches remain available. In our simulations, spiked-in genes are selected independently for each data type; therefore, the comparisons between meta-analytical approaches and single platform analyses are also applicable to situations where different data types are measured in different subjects. Simulation results show that the meta-analytical approach provides improvements over the analysis performed using a single data type, unless several data types present very strong false positive results.

Lastly, application of the integrative approach to two independent glioblastoma datasets has allowed us to discover and validate several gene sets associated with survival. These sets would not receive strong support when expression and CN data are analyzed separately. The role of some of the gene sets found with our approach in GBM survival, such as pathways involved in sugar metabolism, is strongly supported by recent literature. Our suggestion of the previously unreported involvement of the Wnt pathway in GBM survival provides motivation for further, more in-depth biological investigation.

## Materials and methods

### Statistical approaches for multi-platform analysis

#### Notation and problem definition

We begin by defining notation for the single data type case. The data consist of a matrix of genomic measurements X and a vector of phenotypes Y. The genomic measurements are cross-referenced to a membership matrix M whose generic element m_gs _is the indicator of whether gene *g *is in set *s*. Many current gene set analysis methods proceed in two stages [4,36-38]: gene-level testing of differences between groups and set-level testing of differences in scores.

In stage I (gene-level testing of differences between groups), for each gene *g*, compute score *s_g_*(*X*,*Y*), capturing the relationship between genomic measurements and a phenotype.

In stage II (set-level testing of differences in scores), using scores computed in stage I as data, look for association between scores and columns of M. For example, by testing whether the distribution of scores for genes in set *s *is different from the distribution of scores for a reference group, say the set of all measured genes that are not in the set, via a two-sample statistic *t_s_*(*s*,*M_s_*) - the competitive test; or by comparing the observed distribution of *s_g_*(*X*,*Y*), *g *∈ *s*, to its null distribution, when genes are not associated with the phenotype - the self-contained test.

Generalizing the setting of one-dimensional gene set analysis, we assume that we have D dimensions to the genomic investigation. Each dimension is a data type - for example, transcript levels from expression arrays, CN data, somatic mutations, methylation data. We assume that the dimensions are available for the same samples, and that the data provided in each dimension are already summarized by gene.

Data are a series of D matrices of sample-specific genomic measurements *X*^1^,...,*X^D ^*and a vector/matrix of phenotypes Y. These measurements are cross-referenced to a single membership matrix M, as earlier.

#### Integrative approach

Heterogeneous data are integrated into a single gene-specific score, followed by one-dimensional gene set analysis. Formally, stage I consists of evaluating a score *s_g_*(*X*^1^,K,*X^D ^*,*Y*) that draws from all the measurements available from gene *g *across all the dimensions studied.

A relatively simple and general approach is to fit, for every gene, a linear or generalized linear model of the form:

$$\phi \left(E\left(\left(Y_{i}\right|X_{gi}^{1},X_{gi}^{2},...,X_{gi}^{D}\right)\right)=\underset{d\in \left\{1...D\right\}}{\sum }X_{gi}^{d}\beta_{g}^{d}$$

where φ is a link function [39] and *i *the biological sample. For each gene, the stage I score can be a measure of the overall fit of the model, for example, a likelihood ratio for comparing this model to the 'null' model in which all the $\beta_{g}^{d}$ coefficients are zero. In stage II these scores can then be analyzed using traditional methods to obtain set-specific scores *t_s_*(*s*,*M_s_*).

#### Meta-analytical approach

This approach starts as a standard one-dimensional gene set analysis: we determine gene-to-phenotype association scores separately for each dimension and generate a matrix of scores whose generic element is $s_{g}^{d}\left(X^{d},Y\right)$, and in stage II compute set-specific scores $t_{s}^{d}\left(s,M_{s}\right)$,*d *∈ 1,K, *D*, for each dimension. Next these scores can be integrated, say, by averaging:

$$t_{s}\left(s,M_{s}\right)=\underset{d\in \left\{1,...,D\right\}}{\mathrm{avg}}t_{s}^{d}\left(s,M_{s}\right),$$

when evidence of significance from several data types is needed, or by taking an extremum:

$$t_{s}\left(s,M_{s}\right)=\underset{d\in \left\{1,...,D\right\}}{\mathrm{extremum}}t_{s}^{d}\left(s,M_{s}\right),$$

when strong evidence from a single dimension is sought.

### Implementations

#### Single data type analysis

For each gene we compute the gene-to-phenotype association score as the difference of deviances of the logistic regression models with and without the genomic measurement as the single predictor. This score is used in testing for gene sets that are enriched for genes different across phenotypes [4].

#### Integrative approach

For each gene, observations from all available data types are used as independent variables in a multivariate logistic regression model. The integrated gene-to-phenotype association score is computed as the difference of the deviances of the null model and the model with all predictors, and used in gene set tests. We denote this implementation of the integrative approach by INT.

#### Meta-analytical approach

We use geometrically averaged *P*-values, AvgP [40], and minimum *P*-values, MinP [41], from the single-data-type gene set tests.

#### Gene set tests

We use the Mann-Whitney test, as implemented in the R/Bioconductor package *limma *[30], for competitive analysis, and the signed rank Wilcoxon test for self-contained analyses. For the latter we generate the null distribution of gene-to-phenotype association scores by permuting phenotype labels. We perform the test in 500 permutations, comparing the observed scores and null, and then average the resulting test statistics; the *P*-values are obtained from the critical values table of the normal approximation to the Wilcoxon signed rank statistic. We compare discoveries across methods by choosing the same *P*-value cutoff. The *P*-values would remain comparable across methods if one applied a multiple comparison adjustment, as the number of comparisons is the same in the integrative as in each of the single-data-type approaches. While there are many choices for the scores and gene set tests, the simple procedures we chose to implement perform very competitively [4] and adequately represent the general gene set enrichment approach in the context of our proposed integration framework.

### The Cancer Genome Atlas data

We consider TCGA glioblastoma data [2] of four types: two gene expression measurements (E1, E2) using Affymetrix HT-HG-U133A and Agilent G4502A-07 microarrays respectively; and two CN measurements (C1, C2), both using the Agilent HG-CGH-244A platform but performed in two different labs [42]. For expression, we use TCGA's normalization and gene summaries. For CN, we average TCGA-normalized probe values by gene; here we used the probe to gene mapping provided by TCGA in the Array Definition Files. For simulations we use 99 samples with randomly assigned binary phenotypes (49 and 50 observations per class). To discover gene sets important in GBM survival we use an extreme discordant phenotype design [9]. GBM patients with survival time shorter than the lower quartile (190 days) are labeled STSs, and those with survival time longer than the upper quartile (594 days) LTSs. Such grouping enhances signal relevant to survival. After removing patients who developed a hypermutated phenotype due to radiation treatment, we retained 95 observations (47 STS and 48 with LTS phenotypes). There are 12,042 genes for E1, 17,814 for E2, 21,814 for C1, and 23,167 for C2. The total number of genes measured in all data types is 10,334, with 25,583 genes measured in at least one.

For validation, we retrieved expression (Affymetrix U133-Plus-2.0) and CN (Affymetrix 100K-SNP-Array) data for 1,275 genes involved in the pathways discovered in TCGA data analysis (see Results) from the Rembrandt database [8]. Gene summaries are obtained by averaging Rembrandt-preprocessed probe-level data. We used 44 STS samples (survival ≤ 231 days), and 36 LTS samples (survival ≤ 775).

### Simulations

We construct an empirical null scenario, where no genes are associated with the phenotype, by randomly assigning phenotype labels. This preserves correlations between genes within data types, and correlations between data types, yielding a realistic background distribution of gene-to-phenotype association scores. We randomly select ten gene sets from a given gene set collection for 'spiking in'. For each spike-in set we randomly select a fraction of its genes to be associated with the phenotype. The number of genes that differ across phenotypes is sampled from the binomial distribution with parameter γ, which reflects the desired expected fraction of genes in a set to be associated with the phenotype. For each selected gene we add Δk to the observations for one of the phenotypes; Δk is chosen so that, if the data were normal, with standard deviation equal to the average standard deviation among genes in the k-th spiked-in set, then the power for the two-sample *t*-test would have value β. We call β the signal strength.

We study the ability of the methods to discover spiked-in sets as we vary β and γ on a grid. The signal strength captures signal-to-noise ratio: at low values of this parameter, gene-to-phenotype association scores for the spiked-in genes will be more similar to their background null distribution. The expected fraction of altered genes captures enrichment of such genes in a spiked-in set. As this parameter grows, the distribution of gene-to-phenotype association scores within a spiked-in set is more likely to differ from the distribution of the scores for the rest of genes. We use a competitive gene set test to evaluate this difference. Each simulation scenario is repeated 1,000 times. Complete analysis on each simulation repetition takes approximately 10 minutes wall-clock time on a MacBookPro 2.8 GHz Intel Core 2 Duo with 4 GB RAM.

We compute the fraction of correctly discovered spiked-in sets that are discovered with a given data type but not by any of the other data types as:

$$EF\left(i\right)=1-\frac{|{⋃}_{j\in S_{-i}}TP\left(j\right)\cap TP\left(i\right)|}{|TP\left(i\right)|}$$

where *i *indexes data types: *S = {E1,E2,C1,C2}*, *S*_-*i *_is the set of all data types excluding the i-th, and *TP(·) *is the number of true positive sets among the top scoring ones. For each of the three integration methods we also compute the fraction of sets that are discovered exclusively by that method:

$$EF^{*}\left(l\right)=1-\frac{|{⋃}_{j\in S}TP\left(j\right)\cap TP\left(l\right)|}{|TP\left(l\right)|}$$

where *l *∈ {*INT*,*AvgP*,*MinP*}, and *S *and *TP(·) *are defined above.

All computations were performed in the statistical software 'R' [5]. The R code used in our study is available as in Additional file 2. The code and R data objects are also available for download online [43].

## Abbreviations

CN: copy number; GBM: glioblastoma multiforme; INT: model based integration approach; LTS: long-term survivor; MAPK: mitogen-activated protein kinase; MSigDb: Molecular Signatures Database; PI3K: phosphatidylinositol 3-kinase; Rembrandt: Repository for Molecular Brain Neoplasia Data; ROC: receiver operating characteristic; STS: short-term survivor; TCGA: The Cancer Genome Atlas; TNF: tumor necrosis factor, TNFR: tumor necrosis factor receptor.

## Authors' contributions

ST conceived the methodology, designed the analysis plan, developed the software tools and performed all analyses. GP conceived the methodology, designed the analysis plan and provided infrastructural support. LM developed software tools, provided input on cancer biology and biotechnology and contributed to the design of the validation experiments. RK provided input on cancer biology and provided infrastructural support. All authors contributed to the writing. All authors have read and approved the manuscript for publication.

## Supplementary Material

Additional file 1 **Tables with additional results for TCGA GBM data**. Table S1: *P*-values for the top 50 gene sets discovered by the integrative method (INT) using the competitive gene set test. Patients from the upper tertile of the survival distribution were labeled as long-term survivors, and those from the lower tertile short-term survivors. Table S2: *P*-values for the top 50 gene sets discovered by the integrative method (INT) using the competitive gene set test. A Cox regression model was used to establish gene-to-phenotype association scores. Click here for file

Additional file 2 **R code for methods described in the paper**. An archive containing the R implementation of the methods described in the paper, and used for simulations and data analysis. Click here for file
